# SLC25A32 sustains cancer cell proliferation by regulating flavin adenine nucleotide (FAD) metabolism

**DOI:** 10.18632/oncotarget.27486

**Published:** 2020-02-25

**Authors:** Valeria Santoro, Ilya Kovalenko, Kim Vriens, Stefan Christen, Andreas Bernthaler, Andrea Haegebarth, Sarah-Maria Fendt, Sven Christian

**Affiliations:** ^1^ Bayer AG, Drug Discovery, Berlin 13353, Germany; ^2^ Current address: University of Michigan, Cancer Center, Ann Arbor, MI 48108, USA; ^3^ Laboratory of Cellular Metabolism and Metabolic Regulation, VIB Center for Cancer Biology, VIB, Leuven 3000, Belgium; ^4^ Laboratory of Cellular Metabolism and Metabolic Regulation, Department of Oncology, KU Leuven and Leuven Cancer Institute (LKI), Leuven 3000, Belgium

**Keywords:** transporter, mitochondria, metabolism, ROS, FAD

## Abstract

SLC25A32 is a member of the solute carrier 25 family of mitochondrial transporters. SLC25A32 transports tetrahydrofolate (THF) as well as FAD into mitochondria and regulates mitochondrial one-carbon metabolism and redox balance. While it is known that cancer cells require one-carbon and FAD-dependent mitochondrial metabolism to sustain cell proliferation, the role of SLC25A32 in cancer cell growth remains unexplored.

Our results indicate that the *SLC25A32* gene is highly amplified in different tumors and that amplification correlates with increased mRNA expression and reduced patients´ survival. siRNA-mediated knock-down and CRISPR-mediated knock-out of SLC25A32 in cancer cells of different origins, resulted in the identification of cell lines sensitive and resistant to SLC25A32 inhibition. Mechanistically, tracing of deuterated serine revealed that SLC25A32 knock-down does not affect the mitochondrial/cytosolic folate flux as measured by Liquid Chromatography coupled Mass Spectrometry (LC-MS). Instead, SLC25A32 inhibition results in a respiratory chain dysfunction at the FAD-dependent complex II enzyme, induction of Reactive Oxygen Species (ROS) and depletion of reduced glutathione (GSH), which impairs cancer cell proliferation. Moreover, buthionine sulfoximine (BSO) treatment further sensitizes cells to ROS-mediated inhibition of cell proliferation upon SLC25A32 knock-down. Treatment of cells with the FAD precursor riboflavin and with GSH rescues cancer cell proliferation upon SLC25A32 down-regulation.

Our results indicate that the reduction of mitochondrial FAD concentrations by targeting SLC25A32 has potential clinical applications as a single agent or in combination with approved cancer drugs that lead to increased oxidative stress and reduced tumor growth.

## INTRODUCTION

Altered tumor metabolism is described as a hallmark of tumor biology and is essential for the adaptation of tumor cells to their specific needs, e. g. a higher demand for energy and macromolecules [1, 2]. In addition, as a consequence of the activation of specific oncogenes, tumor cells are characterized by a high production rate of ROS and subsequent high anti-oxidative capacity to scavenge damaging levels of oxidative-stress [3].

Due to the glycolytic switch of tumor cells, mitochondrial biology and especially mitochondrial oxidative phosphorylation have been considered of minor importance in cancer biology. However, studies in recent years have provided new insights showing that mitochondrial function is indeed crucial for cancer progression and that oxidative phosphorylation contributes to the majority of cellular ATP within tumor cells [4, 5]. Furthermore, mitochondria are a major source of cellular ROS, which are generated by the mitochondrial respiratory chain mainly in the form of highly reactive superoxide (O_2_^-^) [6]. To avoid tissue damage, mitochondrial superoxide dismutase 2 (SOD2) rapidly converts O_2_^-^ into hydrogen peroxide (H_2_O_2_). However, high levels of H_2_O_2_ can also be damaging to the cell as they form hydroxyl radicals, especially in the presence of iron. The latter have the potential to induce a specific form of cell death by reacting with lipids in cellular membranes. Thus, cells express two main peroxidase systems which are the thioredoxin/peroxiredoxin (TRX/PRX) system and the glutathione/glutathione peroxidase (GSH/GPX) system, both of which oxidize NADPH to NADP^+^ to reduce H_2_O_2_ to H_2_O [7].

Furthermore, mitochondria can regulate diverse metabolic pathways by tightly regulating transport of substrates between the cytosolic and mitochondrial compartments. Although the outer mitochondrial membrane was shown to be relatively permeable, the inner mitochondrial membrane is comparatively impermeable and consequently contains several transporter proteins to overcome such a physical barrier. The SLC25 family (solute carrier family 25) consists of 53 members localized at the inner mitochondrial membrane that transport a wide range of molecules involved in essential mitochondrial processes such as redox balance, the urea and citric acid cycles, oxidative phosphorylation, DNA maintenance and iron metabolism [8]. SLC25A4, A5 A6 and A31 for example transport adenine nucleotides between mitochondria and cytoplasm and are therefore involved in the fine tuning of energy homeostasis [9]. Uncoupling proteins (SLC25A7, A8, A9 A14 and A14) are transporting protons across the mitochondrial membrane and thus, uncouple the transport from ATP generation [10]. Thereby, they are involved in heat production in brown adipose tissue as well as in limiting ROS production from the respiratory chain [11, 12].

SLC25A32 is a mitochondrial transporter and was first characterized by the analysis of Chinese Hamster Ovary (CHO)-gly mutants, mutants of CHO cells that are auxotrophic for glycine [13]. This effect was attributed to a depletion of mitochondrial folate and hence the mutated gene, that was responsible for this phenotype, was proposed to be a mitochondrial transporter of folates [14, 15]. Subsequently, Lawrence et al. applied site-directed mutagenesis to further identify residues within SLC25A32 that are essential for the transport of folate into mitochondria [16].

However, comparisons of human and yeasts orthologues of *SLC25A32* led to the conclusion that SLC25A32 transports FAD/NAD-like substrates [17]. In support of this, yeast lacking the mitochondrial FAD transporter FLX1, could be rescued by human *SLC25A32* expression, suggesting that this transporter may also transport FAD across the inner membrane [18]. In addition to the controversial substrate specificity of SLC25A32, the role of this transporter during tumor progression is entirely uncharacterized. In the present report, we show that *SLC25A32* is highly amplified in a wide range of human tumor samples and that gene amplification correlates with reduced overall survival of cancer patients. Inhibition of SLC25A32 *in vitro* reduces cell proliferation in a subset of tumor cells. In the tumor cell context, this is due to reduced concentrations of FAD in the mitochondria, which leads to a reduction of cellular respiration and an increase in the production of ROS.

Overall, our data suggest that SLC25A32 is an important mitochondrial regulator in cancer cells to maintain mitochondrial FAD levels and that its inhibition represents a potential new strategy to treat cancer by inducing ROS-mediated cancer cell death.

## RESULTS

### SLC25A32 is amplified in human cancer

**Figure 1:**
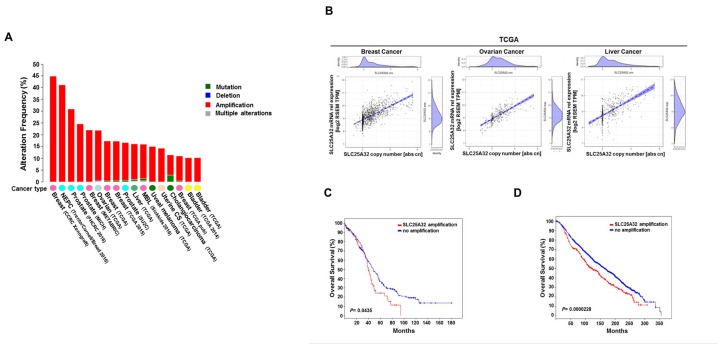
Genetic alterations of SLC25A32 reduce survival of cancer patients. (**A**) Representation of *SLC25A32* genetic alterations across different cancers (www.cbioportal.org). (**B**) Spearman´s rank correlation between SLC25A32 mRNA expression (RSEM TPM) and somatic copy number in breast cancer (1075 sample; *P <* 0.05), ovarian cancer (300 sample; *P =* 0.0.05) and liver cancer (364 sample; *P =* 0.05) in patient samples of TCGA. Each dot represents a tumor sample of one particular patient. The dotted line represents a linear regression line and the blue area around the fitted line shows the 95% confidence intervals. (**C**) Median overall survival data of ovarian carcinoma patients with *SLC25A32* amplification (67 cases) and no amplification (241 cases). Median survival difference between the two groups is statistically significant (*P =* 0.0435). (**D**) Median overall survival data obtained from breast carcinoma patients with *SLC25A32* amplification (407 cases) and no amplification (1459 cases) are presented. Median survival difference between the two groups is statistically significant (*P =* 0.0000228).

To elucidate the role of SLC25A32 in cancer, we used cBioPortal for Cancer Genomics database (www.cbioportal.org) to detect genetic alterations of the *SLC25A32* gene in several human cancers [19, 20]. *SLC25A32* was found to be highly amplified in different tumor types with highest incidence in breast cancer (44.8%), neuroendocrine prostate cancer (30%), ovarian serous cystadenocarcinoma (22%) and liver hepatocellular carcinoma (16.1%) (Figure 1A). Strong correlation between *SLC25A32* amplification and mRNA expression was observed across different tumor types (Supplementary Figure 1) including breast, ovarian and liver cancer (Figure 1B). Furthermore, clinical data showed association between *SLC25A32* amplification and reduced patients´ survival. More specifically, median survival of ovarian cancer patients exhibiting *SLC25A32* gene amplification was 39.85 months as opposed to 48.72 median months survival for patients with no *SLC25A32* amplification (Figure 1C). Similarly, the median survival of breast cancer patients bearing *SLC25A32* amplification was also reduced by 42 months (Figure 1D).

### SLC25A32 knock-down impairs proliferation of different cancer cell lines

**Figure 2:**
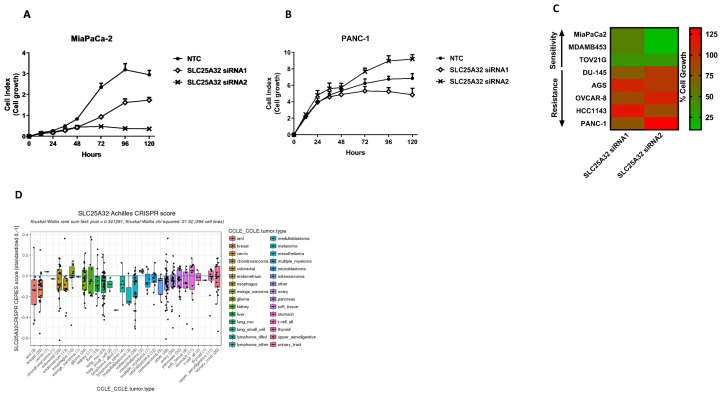
Impairment of SLC25A32 expression inhibits cancer cell proliferation. (**A**, **B**) Cells were transfected with either control siRNA oligo (NTC) or SLC25A32 siRNAs (siRNA1 and siRNA2) and real-time cell proliferation of MiaPaCa-2 (A) and PANC-1 (B) cells was measured with the xCELLigence technology over a period of 5 days. Results are presented as mean ± SD of three biological replicates. (**C**) Heat-map representing effect of SLC25A32 knock-down (96 h time-point) on growth inhibition of MiaPaCa-2, MDAM-MB453, TOV21G, DU-145, AGS, OVCAR-8, HCC1143 and PANC-1 cells. Color green and red define sensitive and resistant cells respectively. Proliferation of cells transfected with SLC25A32 siRNA1 and siRNA2 and measured with xCELLigence technology has been normalized to NTC sample and presented as % of cell growth. (**D**) Effect of CRISPR-mediated SLC25A32 knock-out on proliferation of 394 cancer cell line (Achilles Project, Broad Institute). Negative CERES scores indicate more sensitive cell lines.

To investigate the role of SLC25A32 as a potential cancer target we assessed the effect of SLC25A32 knock-down on the proliferation of a panel of tumor cell lines of different origins (Supplementary Figure 2A). To this end, eight cancer cell lines were transfected with two different siRNA oligos targeting SLC25A32 and one non-targeting control oligo (NTC). Inhibition of cell proliferation was subsequently measured over time. While both siRNAs strongly reduced SLC25A32 mRNA levels in all cancer cell lines analyzed (Supplementary Figure 2B, 2C), the effects exhibited on cell proliferation were different. SLC25A32 siRNA1 and more strongly siRNA2 inhibited cell proliferation of MiaPaCa-2 (Figure 2A), MDA-MB453 (Supplementary Figure 2D) and TOV21G (Supplementary Figure 2E), as measured by the impedance-based real-time cell analyzer (RTCA). In contrast, no inhibitory effects on cell growth were observed in PANC-1 (Figure 2B), AGS (Supplementary Figure 2F), OVCAR-8 (Supplementary Figure 2G), DU-145 (Supplementary Figure 2H) and HCC1143 (Supplementary Figure 2I) cells upon SLC25A32 knock-down with either siRNA.

Overall, the effects of SLC25A32 inhibition on the proliferation of eight cancer cell lines are summarized in Figure 2C and indicate a clear separation between sensitive (cell growth normalized to NTC control <50%) and resistant cell lines (cell growth normalized to NTC control >75%).

Moreover, data from a genome-scale CRISPR-Cas9 loss-of-function screen (Project Achilles) was also used to corroborate our siRNA-based findings [21]. The impact of CRISPR-mediated SLC25A32 knock-out on the viability of 394 cancer cells is represented in Figure 2D. Among the eight cancer cell lines used in this study (Figure 2C), six were also found to be part of the Achilles CRISPR screen. Sensitive (MiaPaCa-2, MDA-MB453 and TOV21G) and resistant cell lines (AGS, OVCAR-8 and HCC143) are highlighted in green and red respectively and their response to SLC25A32 CRISPR-KO correlates with the effects observed in our siRNA-based dataset (Figure 2D). Lower values of CERES scores indicate more sensitive cell lines (Figure 2D).

### SLC25A32 knock-down does not affect the folate cycle in MiaPaCa-2 and in PANC-1 cells

**Figure 3:**
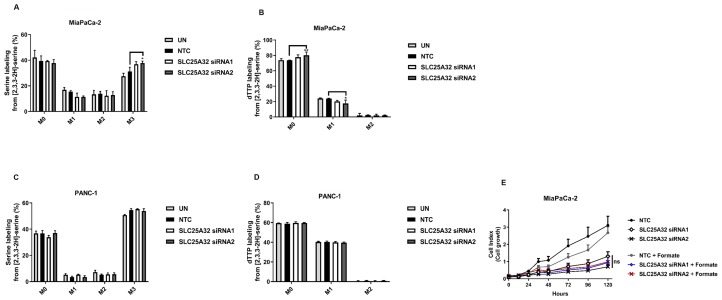
SLC25A32 knock-down does not impair folate cycle activity. (**A**) [2,3,3-^2^H]-serine isotopomer distribution in MiaPaCa-2 cells transfected with either control siRNA oligo (NTC) or SLC25A32 siRNAs (siRNA1 and siRNA2) for 48 h. UN: untreated cells. Results are presented as mean ± SD (*n=3*), M3 NTC vs. SLC25A32 siRNA2 ^*^ *P =* 0.0318. (**B**) [2,3,3-^2^H]-serine labeling into dTTP in MiaPaCa-2 transfected as in A. Results are presented as mean ± SD (*n = 3*), M0 NTC vs. SLC25A32 siRNA2 (^**^ *P =* 0.0092); M1 NTC vs. SLC25A32 siRNA2 (^**^ *P =* 0.0113). (**C**) [2,3,3-^2^H]-serine isotopomer distribution in PANC-1 cells transfected as in (A). Results are presented as mean ± SD (*n = 3*). (**D**) [2,3,3-^2^H]-serine labeling into dTTP in PANC-1 cells transfected with either control siRNA oligo (NTC) or SLC25A32 siRNAs (siRNA1 and siRNA2) for 48 h. Results are presented as mean ± SD (*n = 3*). (**E**) Cells were transfected with either control siRNA oligo (NTC) or SLC25A32 siRNAs (siRNA1 and siRNA2) and real-time cell proliferation of MiaPaCa-2 cells was measured with the xCELLigence technology. Cells were treated with Formate (1 mM) for rescue experiment. Results are presented as mean ± SD (*n = 3*). (A–E) Statistical analysis was conducted with Graphpad PRISM (Two-way ANOVA; Bonferroni post-test). For graphs A–D, only comparisons showing statistical significance are represented.

SLC25A32 has been previously described as a mitochondrial transporter of THF [16]. We therefore assessed the impact of its down-regulation on the compartmentalization of one-carbon folate cycle in one sensitive (MiaPaCa-2) and one resistant (PANC-1) cell line. Previous studies have shown that upon loss of function of mitochondrial folate enzymes, certain cancer cells reverse the cytosolic one-carbon metabolism flux to sustain growth [22]. In order to directly track mitochondrial and cytosolic one-carbon unit production, cells were fed with [2,3,3-^2^H]-serine and subsequent labeling of thymidine triphosphate (dTTP) was measured via LC-MS upon 48 and 72 h SLC25A32 knock-down. Cytosolic production of thymidine directly from methylene-THF via SHMT1 enzyme (reversal of cytosolic flux) would lead to M+2 dTTP labelling. In contrast, transfer of one carbon units from the mitochondria to the cytosol would generate M+1 dTTP. Our data show that treatment of MiaPaCa-2 and PANC-1 cells with [2,3,3-^2^H]-serine resulted in serine M+3 label incorporation in control and SLC25A32 knock-down samples at 48 h (Figure 3A, 3C) and 72 h (Supplementary Figure 3A, 3C) post-transfection. When measuring dTTP labeling from serine, we observed M+1 labeled dTTP in both MiaPaCa-2 and PANC-1 cells and no increase in M+2 dTTP in either control samples or SLC25A32 knock-down samples at 48 h (Figure 3B, 3D) and 72 h time-points (Supplementary Figure 3B, 3D). Furthermore PANC-1 cells show higher basal levels of M+1 labelled dTTP when compared to MiaPaCa-2 cells, indicating a higher activity of the mitochondrial folate pathway in this cell line (Figure 3B, 3D and Supplementary Figure 3B, 3D). Overall, our results indicate that both cell lines exhibit an active mitochondrial folate pathway and that SLC25A32 knock-down does not induce reversal of the cytosolic cycle. In support of this evidence, addition of extracellular formate (1 mM) did not rescue the proliferation of MiaPaCa-2 cells upon SLC25A32 down-regulation (Figure 3E).

### Riboflavin rescues cell proliferation and respiratory chain defect at complex II upon SLC25A32 down-regulation

**Figure 4:**
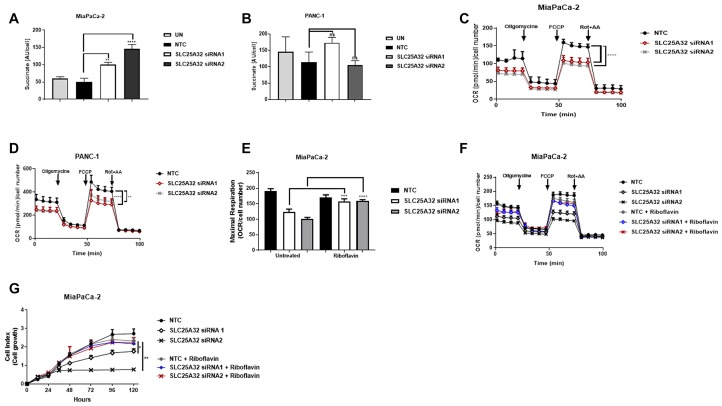
SLC25A32 knock-down impairs mitochondrial FAD metabolism. (**A**, **B**) Succinate levels were measure by LC-MS following 72 h SLC25A32 knock-down with siRNA1 and siRNA2 in MiaPaCa-2 (A) and PANC-1 (B) cells. Results for non-targeting control (NTC) and untreated (UN) cells are also presented. Succinate levels were normalized to cell number. (A) NTC vs. SLC25A32 siRNA1, ^**^ *P =* 0.0013; NTC vs. SLC25A32 siRNA2, ^****^ *P* ≤ 0.0001. (**C**, **D**) Oxygen consumption rate (OCR) of MiaPaCa-2 (C) and PANC-1 (D) cells was measured with the Seahorse XF Analyzer following 72 h transfection with non-targeting control (NTC) siRNA, SLC25A32 siRNA1 and SLC25A32 siRNA1. Mitochondrial activity was assessed by consecutive injections of oligomycin (1 μM), FCCP (0.5 μM), and antimycin A (1 μM) or rotenone (1 μM). Results were normalized to cell number and presented as mean ± SD (*n = 3*), (C) NTC vs. SLC25A32 siRNA1, ^****^ *P ≤* 0.0001; NTC vs. SLC25A32 siRNA2, ^****^ *P ≤* 0.0001, (D) NTC vs. SLC25A32 siRNA1, ^**^ *P =* 0.0013; NTC vs. SLC25A32 siRNA2, ^****^ *P ≤* 0.0001. (**E**) OCR measurement in MiaPaCa-2 cells was conducted as in C. Cells were treated with Riboflavin (10 μM) for rescue experiment. SLC25A32 siRNA1 untreated vs. riboflavin, ^***^ *P =* 0.0013; SLC25A32 siRNA2 untreated vs. riboflavin, ^****^ *P ≤* 0.0001. (**F**) Bar graph of maximal respiration capacity from experiment conducted in E. Data presented is extracted from second time-point following FCCP injection. Results were normalized to cell number and presented as mean ± SD (*n = 3*). (**G**) Cells were transfected with either control siRNA oligo (NTC) or SLC25A32 siRNAs (siRNA1 and siRNA2) and real-time cell proliferation of MiaPaCa-2 cells was measured with the xCELLigence technology. Cells were treated with Riboflavin (10 μM) for rescue experiment. Results are presented as mean ± SD (*n = 3*). SLC25A32 siRNA1 untreated vs. riboflavin, ^*^ *P =* 0.0217; SLC25A32 siRNA2 untreated vs. riboflavin, ^**^ *P =* 0.0031. (A–G) Two-way ANOVA and Bonferroni post-test were used for statistical analysis with Graphpad-Prism6.

Results presented in Figure 3 indicate that the folate cycle is not disrupted by SLC25A32 knock-down and therefore substrates other than THF could be transported into the mitochondria by SLC25A32. In support of this hypothesis, work from Spaan *et al*. showed that expression of human *SLC25A32* rescued the growth of a yeast strain defective for Flx1 protein (FAD transporter) [18]. We therefore assessed the activity of the FAD-dependent mitochondrial enzyme Succinate Dehydrogenase (Complex II) and mitochondrial respiration as a measure of FAD-dependent mitochondrial function. Interestingly, inhibition of SLC25A32 expression resulted in increased succinate levels in sensitive MiaPaCa-2 cells suggesting a defect at complex II upon SLC25A32 knock-down (Figure 4A). In contrast, succinate levels did not significantly change in PANC-1 resistant cells upon SLC25A32 down-regulation (Figure 4B). To further assess impairment of mitochondrial function, oxygen consumption rate (OCR) was measured with the Seahorse XF Analyzer. SLC25A32 knock-down reduced basal OCR in both MiaPaCa-2 and PANC-1 cells (Figure 4C, 4D). Maximum respiration capacity, as measured after injection of the mitochondrial uncoupler FCCP, was also strongly reduced in both cell lines tested (Figure 4C, 4D). Furthermore, treatment with the FAD precursor Riboflavin rescued maximal respiration capacity in MiaPaCa-2 cells by 40% ± 8% and by 60% ± 4% upon siRNA1 and siRNA2-mediated SLC25A32 knock-down respectively (Figure 4E, 4F). In accordance with these findings, Riboflavin could also rescue the proliferation of MiaPaCa-2 cells upon SLC25A32 down-regulation (Figure 4G).

### Reactive Oxygen Species (ROS) production is induced upon SLC25A32 down-regulation

**Figure 5:**
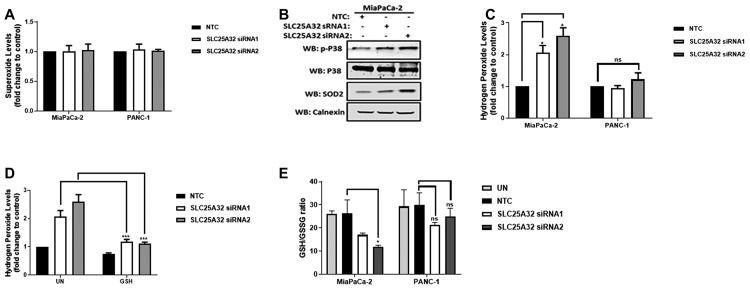
SLC25A32 knock-down induces ROS production and GSH depletion in MiaPaCa-2 cells. (**A**) MiaPaCa-2 and PANC-1 cells were transfected with siRNAs targeting SLC25A32 (siRNA1 and 2) and a non-targeting control (NTC) for 72 h. Cells were stained with Mitosox red dye (5 μM) for 30 min and superoxide levels were detected by flow cytometry. Results are presented as mean ± SD, relative to NTC. MiaPaCa-2 cells, NTC vs. SLC25A32 siRNA 1, ^*^ *P =* 0.0494 and NTC vs. SLC25A32 siRNA 2, ^*^ *P =* 0.0224. (**B**) MiaPaCa-2 cells were transfected as in (A). Proteins were extracted and resolved by Immunoblotting. Membranes were incubated with the indicated antibodies. Calnexin was used as loading control. (**C**) MiaPaCa-2 and PANC-1 cells were transfected as in (A). Cells were stained with H_2_DCFDA dye (5 μM) for 30 min and hydrogen peroxide levels were detected by flow cytometry. Results are presented as mean ± SD, relative to NTC. (**D**) MiaPaCa-2 were transfected as in (A). Cells were treated with cell permeable glutathione (GSH 100 μM) and stained with H_2_DCFDA dye (5 μM) for 30 min and hydrogen peroxide levels were detected by flow cytometry. Results are presented as mean ± SD, relative to NTC. SCLC25A32 siRNA 1 untreated vs. GSH, ^***^ *P =* 0.0001 and SCLC25A32 siRNA 2 untreated vs. GSH, ^***^ *P =* 0.0001. (**E**) MiaPaCa-2 cells were transfected as in A. Ratio of reduced (GSH) to oxidized glutathione (GSSG) was measured by LC-MS. Results are presented as mean ± SD (*n=3*). MiaPaCa-2 NTC vs. SLC25A32 siRNA2, ^*^ *P =* 0.0201. (A–E) Two-way ANOVA and Bonferroni post-test were used for statistical analysis with Graphpad-Prism6.

ROS are produced at the Electron Transport Chain (ETC) when electron transfer at the ETC is disrupted or when intracellular antioxidant systems are depleted or defective. We therefore sought to investigate whether impaired mitochondrial function of MiaPaCa-2 and PANC-1 cells could lead to increased ROS production. To this end, mitochondrial superoxide levels were measured by MitoSOX red dye and FACS analysis. Interestingly, no superoxide increase could be detected upon 72 h SLC25A32 knock-down in both MiaPaCa-2 and PANC-1 cells (Figure 5A). Furthermore, SLC25A32 knock-down induced activation of stress-activated kinase p38 as well as up-regulation of mitochondrial superoxide dismutase (SOD2) protein (Figure 5B). We therefore used the H_2_DCFDA dye to detect hydrogen peroxide which is a product of superoxide dismutation in the mitochondria and that can freely cross cellular membranes. Hydrogen peroxide levels were significantly increased in MiaPaCa-2 cells by 1 ± 0.23 and 1.5 ± 0.26 fold upon SLC25A32 siRNA1 and siRNA2 transfection respectively (Figure 5C). In contrast, no significant hydrogen peroxide generation was detected in resistant PANC-1 cells (Figure 5C). Moreover, SLC25A32 siRNA1 and siRNA2-mediated ROS production was significantly reduced by GSH treatment from 2 ± 0.23 to 1.1 ± 0.01 fold and from 2.5 ± 0.26 to 1.1 ± 0.05 fold respectively (Figure 5D). We subsequently assessed the ratio of GSH (reduced form of glutathione) to GSSG (oxidized form) as a measure of cellular stress and antioxidant capacity. Indeed, the ratio of reduced to oxidized glutathione was significantly reduced in SLC25A32 knock-down samples when compared to non-targeting control in MiaPaCa-2 cells but not in PANC-1 cells (Figure 5E). Total cellular GSH and GSSG levels measured by LC-MS in both MiaPaCa-2 and PANC-1 cells are presented in Supplementary Figure 4A, 4B.

### SLC25A32 knock-down induces ROS-mediated inhibition of cancer cell proliferation

**Figure 6:**
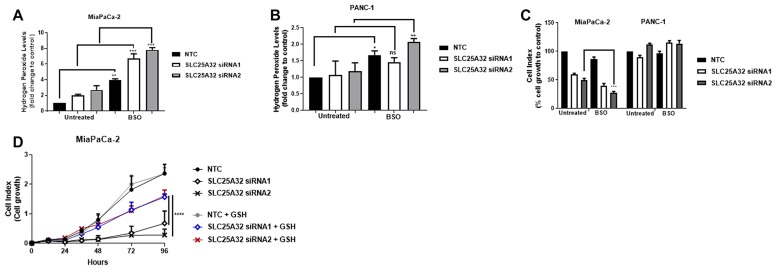
ROS-inducer BSO further sensitizes MiaPaCa-2 cells to SLC25A32 inhibition. (**A**, **B**) MiaPaCa-2 (A) and PANC-1 (B) cells were transfected with siRNAs targeting SLC25A32 (siRNA1 and 2) and a non-targeting control (NTC) for 72 h and treated with L-Buthionine sulfoximine (BSO 10 μM). Cells were stained with H_2_DCFDA dye (5 μM) for 30 min and hydrogen peroxide levels were detected by flow cytometry. Results are presented as mean ± SD, relative to NTC. (A) NTC untreated vs. BSO, ^**^ *P =* 0.0030, SLC25A32 siRNA1 untreated vs. BSO, ^***^ *P =* 0.0002, SLC25A32 siRNA2 untreated vs. BSO, ^***^ *P =* 0.0001**;** (B) NTC untreated vs. BSO, ^*^ *P =* 0.0385, SLC25A32 siRNA2 untreated vs. BSO, ^***^ *P =* 0.0050. (**C**) Cell growth of MiaPaCa-2 and PANC-1 cells transfected with siRNAs targeting SLC25A32 (siRNA1 and 2) and a non-targeting control (NTC) was measured with the XCELLigence technology. At the time of transfection, BSO 10 μM was added to cells. Data from the 96 h time-point were normalized to NTC control and presented as mean ± SD (*n = 3*). MiaPaCa-2, SLC25A32 siRNA2 untreated vs. BSO, ^***^ *P =* 0.0002. (**D**) Cell growth of MiaPaCa-2 cells transfected with siRNAs targeting SLC25A32 (siRNA1 and 2) and a non-targeting control (NTC) was measured with the XCELLigence technology. At the time of transfection, reduced glutathione (GSH 100 μM) was also added to cells. Results are normalized to NTC control and presented as mean ± SD (*n = 3*). SLC25A32 siRNA1 untreated vs. GSH, ^****^ *P* ≤ 0.0001, SLC25A32 siRNA2 untreated vs. GSH, ^****^ *P* ≤ 0.0001. (A–D) Two-way ANOVA and Bonferroni post-test were used for statistical analysis with Graphpad-Prism6.

To investigate whether combined inhibition of glutathione synthesis and SLC25A32 expression would result in a synergistic effect on ROS production and inhibition of cell proliferation, SLC25A32-KD cells were treated with Buthionine Sulphoximine (BSO), an inhibitor of the rate-limiting step of GSH synthesis. Treatment of MiaPaCa-2 cells with 10 μM BSO further increased ROS levels by 3 ± 0.15, 6 ± 0.62 and 7 ± 0.13 fold in non-targeting control, SLC25A32 siRNA1 and siRNA2 samples respectively (Figure 6A). In contrast, treatment of PANC-1 cells with the same concentration of BSO resulted in only a minor ROS increase upon SLC25A32 knock-down (Figure 6B). Moreover, BSO treatment further impaired proliferation of MiaPaCa-2 cells by an additional 20% ± 4.09 and by 30% ± 2.1 upon SLC25A32 siRNA1 and siRNA2 knock-down respectively (Figure 6C). In contrast, no significant effect on cell proliferation was observed in PANC-1 cells (Figure 6C). Ultimately, the antioxidant GSH was able to rescue proliferation of MiaPaCa-2 cells transfected with SLC25A32 siRNA1 and siRNA2 (Figure 6D). Overall our results indicate that GSH depletion and ROS production further inhibit proliferation of MiaPaCa-2 cells upon SLC25A32 down-regulation.

## DISCUSSION

Our work highlights the importance of SLC25A32 as a novel regulator of cancer cell proliferation and mitochondrial FAD metabolism, since until now its function remains unknown in the field of cancer research. We show that *SLC25A32* is highly amplified in different tumor types and that gene amplification correlates with increased mRNA expression and reduced patients´ survival, emphasizing its prominent clinical relevance and high potential as a novel target for cancer therapy. This is further underlined by loss-of function experiments using siRNA or CRISPR that reveal a significant impact on tumor cell proliferation in a subset of human tumor cell lines.

Studies have shown that in CHO cells, SLC25A32 regulates folate metabolism via the transport of THF into mitochondria [16, 15]. Despite the lack of published reports directly linking SLC25A32 to cancer, the role of one-carbon and folate metabolism in cancer cell survival is well established. It is known that several cancer cells highly express *de novo* serine synthesis or mitochondrial folate pathway genes and that their expression is essential for the maintenance of the malignant phenotype [23–27]. We therefore assessed SLC25A32-dependent regulation of the folate 1-C flux by adopting the [2,3,3-^2^H]-serine tracing method from Ducker *et al*. [22]. It has been shown that CRISPR-mediated gene knock-out of mitochondrial folate pathway genes in HEK-293, prostate LNCaP and pancreatic 8988T cells enabled reversal of the cytosolic flux in nutrient-replete conditions and dependence on extracellular serine and glycine [22]. Interestingly, our data indicate that inhibition of SLC25A32 does not reduce the activity of the mitochondrial (M+1 dTTP) folate cycle and does not induce reversal of the cytosolic flux (M+2 dTTP) in neither sensitive nor resistant tumor cells. Furthermore, in contrast to published reports showing cancer cell dependency on mitochondrial folate enzymes only in the absence of extracellular serine, the knock-down of SLC25A32 in nutrient-replete conditions strongly inhibited proliferation of sensitive tumor cell lines *in vitro*. Our findings therefore suggest that SLC25A32 down-regulation does not alter the mitochondrial/cytosolic folate cycle and that cancer cell dependency on SLC25A32 expression might be driven by other pathways than serine-derived mitochondrial one-carbon metabolism.

Interestingly, the work from Spaan *et al*. indicates that SLC25A32 is the human orthologue of the yeast mitochondrial FAD transporter, since growth of FLX1 (FAD transporter)-mutant yeast strain was restored by overexpression of human *SLC25A32* [18]. Our data support the role of SLC25A32 as a mitochondrial regulator of FAD levels and show that SLC25A32 knock-down in sensitive tumor cells results in defect at the FAD-dependent complex II enzyme, increased succinate levels and reduced OCR. Interestingly, SLC25A32 knock-down did not induce succinate accumulation in PANC-1 resistant cells while impaired respiration could indeed be observed. Since PANC-1 cells exhibit significantly higher basal levels of succinate and OCR when compared to sensitive MiaPaCa-2 cells, it is possible that mitochondrial FAD concentrations in these cells are considerably higher and thus are able to sustain sufficient FAD levels for a longer time period. Furthermore, treatment with the FAD precursor Riboflavin could completely rescue oxygen consumption and proliferation of sensitive tumor cells, reinforcing our results linking reduced mitochondrial FAD concentrations and inhibition of cancer cell proliferation. In support of our data, exome sequencing conducted on patients with Multiple acyl-coenzyme A dehydrogenation deficiency (MADD) or with a severe neuromuscular phenotype, revealed novel variant mutations in the SLC25A32 gene [28, 29]. In these studies both Complex II protein levels and activity of OXPHOS, used as a marker for mitochondrial FAD in muscle biopsies, were reduced in patients bearing SLC25A32 mutations [29, 28]. In addition, oral administration of Riboflavin could improve the exercise intolerance of these patients and improve their biochemical phenotype of FAD deficiency.

Impaired mitochondrial FAD transport can also affect other pathways important for cancer cell survival. These include the regulation of ROS production and scavenging as well as fatty acid oxidation reactions. Cancer cells are highly dependent on ROS-scavenging systems in order to balance their high ROS production rate [30]. The activity of glutathione reductase (GR), the enzyme that regenerates reduced and active GSH from its oxidized form GSSG, is not only NADPH-dependent but also FAD-dependent. Defects in the respiration machinery upon SLC25A32 knock-down result in high levels of hydrogen peroxide and in increased GSSG levels only in sensitive MiaPaCa-2 cells. Interestingly, increased SOD2 expression upon SLC25A32 knock-down enhanced the dismutation of superoxide into hydrogen peroxide. Reports have shown that SOD2 expression can be increased in response to stress via the activation of various transcription factors (NRF, p53, AP1, nuclear factor (NF)-κB etc.) [31]. In contrast, resistant cells did not show any alteration in ROS production and GSH turnover upon SLC25A32 knock-down, suggesting a higher activity or expression of other antioxidant systems in these cells. In support of this, our data show that PANC-1 cells exhibit higher basal levels of M+1 dTTP labelling when compared to MiaPaCa-2 cells and, therefore, ROS levels might be more easily scavenged due to increased one-carbon metabolism in these cells. In fact, mitochondrial one-carbon reactions are important for the production of glycine, precursor of GSH, and for the generation of the antioxidant cofactor NADPH [32, 33].

Treatment with BSO, an inhibitor of the rate-limiting step of GSH synthesis, was used to determine the reliance of sensitive and resistance cells on glutathione-dependent antioxidant systems for cell survival. Our results indicate that BSO synergizes with SLC25A32 knock-down in inhibiting cell proliferation and producing ROS only in SLC25A32-sensitive tumor cells. Therefore, it is likely that sensitive and resistant cells differ in their antioxidant capacities. High ROS levels induce destabilization of KEAP1 and stabilization of NRF2 protein [34], the transcriptional regulator of genes involved in GSH synthesis, and in reduction of oxidized GSH and thioredoxin (TXN) systems [30, 35]. Moreover, it has been shown that the antioxidant response also relies on the flux of glucose- and glutamine-derived carbons to produce antioxidant cofactors such as NADP/NADPH [36, 37]. Consequently, the activation and the preferred usage of such pathways can differ among different cancer cells.

Furthermore, it is likely that higher concentrations of BSO (>10 μM) or treatment with other pro-oxidants acting on other pathways than GSH-dependent ones (e. g. hydrogen peroxide), could be necessary to increase the ROS levels above the tolerated threshold present in resistant cells. In support of our work proposing SLC25A32 as novel cancer target regulating oxidative-stress metabolism, other studies have also shown the potential of ROS-inducing therapies as effective anti-cancer strategies [38–40, 35].

Overall our data suggest that inhibition of SLC25A32 is anti-proliferative in a subset of tumor cell lines, at least partially by an increase of reactive oxygen species as a result of a malfunctional FAD-dependent enzymes such as SDH and that resistant cell line can compensate for the loss by the availability of higher reducing capacities. The study validates the role of SLC25A32 as a novel cancer target involved in the regulation of FAD-dependent mitochondrial metabolism. Molecular targeting of SLC25A32 using a single agent or in combination with ROS-inducing therapies could be an effective clinical strategy to successfully treat cancer patients.

## MATERIALS AND METHODS

### Cell lines and culture conditions

MiaPaCa-2, MDAMB-453, AGS, OVCAR-8 and DU-145 cells were cultured in DMEM/F12 with 10% fetal calf serum; PANC-1 cells were cultured in DMEM with 10% fetal calf serum; TOV21G cells were cultured in 50% MCDB 153 + 50% Medium 199 with 10% fetal calf serum and 2 mM L-Alanyl-L-Glutamine; HCC1143 were cultured with RPMI 1640 with 20% fetal calf serum. All cell media were supplemented with 2 mM glutamax.

### Reagents

Cell permeable glutathione (GSH), L-Buthionine-sulfoximine (BSO), Riboflavin and Sodium Formate were purchased from Sigma-Aldrich (St. Louis, MO, USA). [2,3,3-^2^H]-Serine tracer was purchased from Buchem BV (Apeldoorn, Netherlands), Lipofectamine RNAiMAX from Life Technologies (Carlsbad, CA, USA) and non-targeting siRNA (scrambled control) and SLC25A32 siRNAs were ordered from Qiagen (Hilden, Germany).

### siRNA transfection

2–4 × 10^5^ cells/well were seeded in 6 well plates and transfected with siRNAs (10 nM) according to Lipofectamine RNAiMAX (Life Technologies, Carlsbad, CA, USA) protocol.

### Real-time PCR analysis

Total RNA was extracted from cells upon siRNA transfection with NucleoSpin RNA Mini (Macherey-Nagel, Düren, Germany) kit and the QiaCube extraction System (Qiagen, Hilden, Germany) according to manufacturer’s protocol. Template cDNA was generated from 1 μg RNA using the Maxima Reverse Transcriptase enzyme kit (Life Technologies, Carlsbad, CA, USA) and PCR was performed using the TaqMan^®^ Fast Advanced Master Mix according to manufacturer´s instructions. TaqMan primers were purchased from Life Technologies (SLC25A32; Assay ID: Hs00229219_m1; catalog # 4331182 and HPRT1; Assay ID: Hs028000695_m1; catalog # 4331182).

### Immunoblotting

Cells were harvested and proteins were extracted in RIPA buffer (Life Technologies, Carlsbad, CA, USA) supplemented with complete Mini EDTA-free protease inhibitors (Roche Life Sciences, Indianapolis, IN, USA) and PhosphoSTOP phosphatase inhibitors (Roche Life Sciences, Indianapolis, IN USA). BCA Protein Assay was used to measure protein concentration (Life Technologies, Carlsbad, CA, USA) according to manufacturer´s instructions. Detailed methods are provided in the Supplementary Methods.

### Proliferation assay

200 cells/well were seeded in the xCELLigence RTCA MP 96 well plates and reverse transfection with siRNAs (10 nM) was conducted according to Lipofectamine RNAiMAX (Life Technologies, Carlsbad, CA, USA) protocol. Cell growth was measured real time using xCELLigence RTCA MP Station (ACEA Biosciences, San Diego, CA, USA) over a period of 5 days. Riboflavin (10 μM), GSH (100 μM), BSO (10 μM) and Sodium Formate (1 mM) were added at the time of transfection.

### ROS detection

Superoxide and Hydrogen Peroxide levels were measured with MitoSOX Red dye (Life Technologies, Carlsbad, CA, USA) and with H_2_DCFDA dye respectively (Life Technologies, Carlsbad, CA, USA). 72 hours post-transfection with siRNAs, cells were washed with PBS, and incubated with either MitoSOX Red or H_2_DCFDA dye (5 μM) for 30 min. Subsequently, cells were trypsinized and analyzed by Fluorescence-Activating Cell Sorting (FACS) (Beckman Coulter, Brea, CA, USA). GSH (100 μM) or BSO (10 μM) were added at time of transfection.

### LC-MS measurements and data analysis

150.000 cells/well (MiaPaCa-2) and 100.000 cells/well (PANC-1) were seeded in 6-well plates and the following day cells were transfected with SLC25A32 siRNAs. Upon 24 h knock-down, cells were washed with DBPS and incubated with fresh labeling medium (3 mL/well) for 24 h and 48 h:

-MiaPaCa-2: DMEM/F12/Glutamax; 10% dialysed FBS; 250 μM [2,3,3-^2^H]-serine on top, resulting in a final concentration of 500 μM serine and 50% label).

-PANC-1: DMEM; 10% dialysed FBS; 400 μM [2,3,3-^2^H]-serine on top, resulting in a final concentration of 800 μM serine and 50% label).

Afterwards, cell metabolism was quenched by flash freezing the plates in liquid nitrogen and metabolites were extracted as previously described [41]. Extracted polar metabolites were dried overnight at 4° C and dissolved in 60% acetonitrile in water prior to LC-MS measurements. Chromatography and MS measurements were performed with an Agilent 1290 Infinity HPLC coupled to a 6470 Triple quadrupole instrument. All metabolites, except for nucleotides, were separated on a iHILIC column with buffer A 100% acetonitrile and buffer B 10 mM ammonium acetate pH 9.3 in water. Nucleotides were separated on a iHILIC column with buffer A 100% acetonitrile and buffer B 20 mM ammonium acetate pH 9.3 in water. Peak integration was performed with MassHunter software provided by Agilent for LC-MS samples. For sample comparison, the data were normalized to cell number.

### Seahorse XF-96 analyzer

10.000–15.000 cells/well were seeded in Seahorse 96 well plates in growth media. The next day, cells were transfected with 10 nM siRNAs and with Lipofectamine RNAiMAX reagent according to manufacturer´s instructions. 72 h post-transfection cells were washed twice with PBS (180 μL/well) and incubated for the duration of the assay with DMEM seahorse media (180 μL/well) supplemented with 1.5g/l NaCl, 5 mM glucose, 2 mM glutamine at pH = 7.4, 37°. Mitochondrial stress kit was employed and compounds used to characterize mitochondrial function such as Oligomycin (1 μM), FCCP (0.5 μM), Rotenone (1 μM) and Antimycin A (1 μM) were injected at fixed intervals every four measurements. Cellular oxygen consumption rate (OCR) was normalized to cell number.

### TCGA and Achilles CRISPR data analysis

TCGA RNASeq (RSEM TPM) and somatic copy number (derived on Affymetrix SNP6.0) data was obtained using FireBrowser [42]. The data is available on https://gdac.broadinstitute.org/. Achilles CRISPR data has been taken as provided by the Achilles consortium and can freely be accessed [21]. In brief, the authors perform basic preprocessing and quality control of the screening data (including deconvolution of raw barcode counts from the sequencing data) and computationally correct for anti-proliferative effects of Cas9-mediated DNA cleavage using the CERES algorithm. Then, data is normalized in such that a CERES score of 0 of a particular gene represents effects of previously defined non-essential genes, and a CERES score of -1 represents effects of previously defined essential genes as described [43].

### Statistical analysis

One-way or Two-way ANOVA statistical test were used and analysis was performed using GraphPad Prism software version 6.0 (GraphPad Software, La Jolla, CA, USA). Statistical significance is indicated as follows: Statistical significance is indicated as follows: ^*^for *P* < 0.05, ^**^ for *P* < 0.01; ^***^ *P* < 0.001 and ^****^ for *P* < 0.0001.

## SUPPLEMENTARY MATERIALS


