# Supplementary material for: SLC25A32 sustains cancer cell proliferation by regulating flavin adenine nucleotide (FAD) metabolism
Source: Oncotarget. 2020 Feb 25;11(8):801–12. doi: 10.18632/oncotarget.27486 (PMC7055544; doi:10.18632/oncotarget.27486)
Supplement: Supplementary file 1 [file oncotarget-11-801-s001.pdf]

SLC25A32 sustains cancer cell proliferation by regulating flavin adenine nucleotide (FAD) metabolism

SUPPLEMENTARY MATERIALS

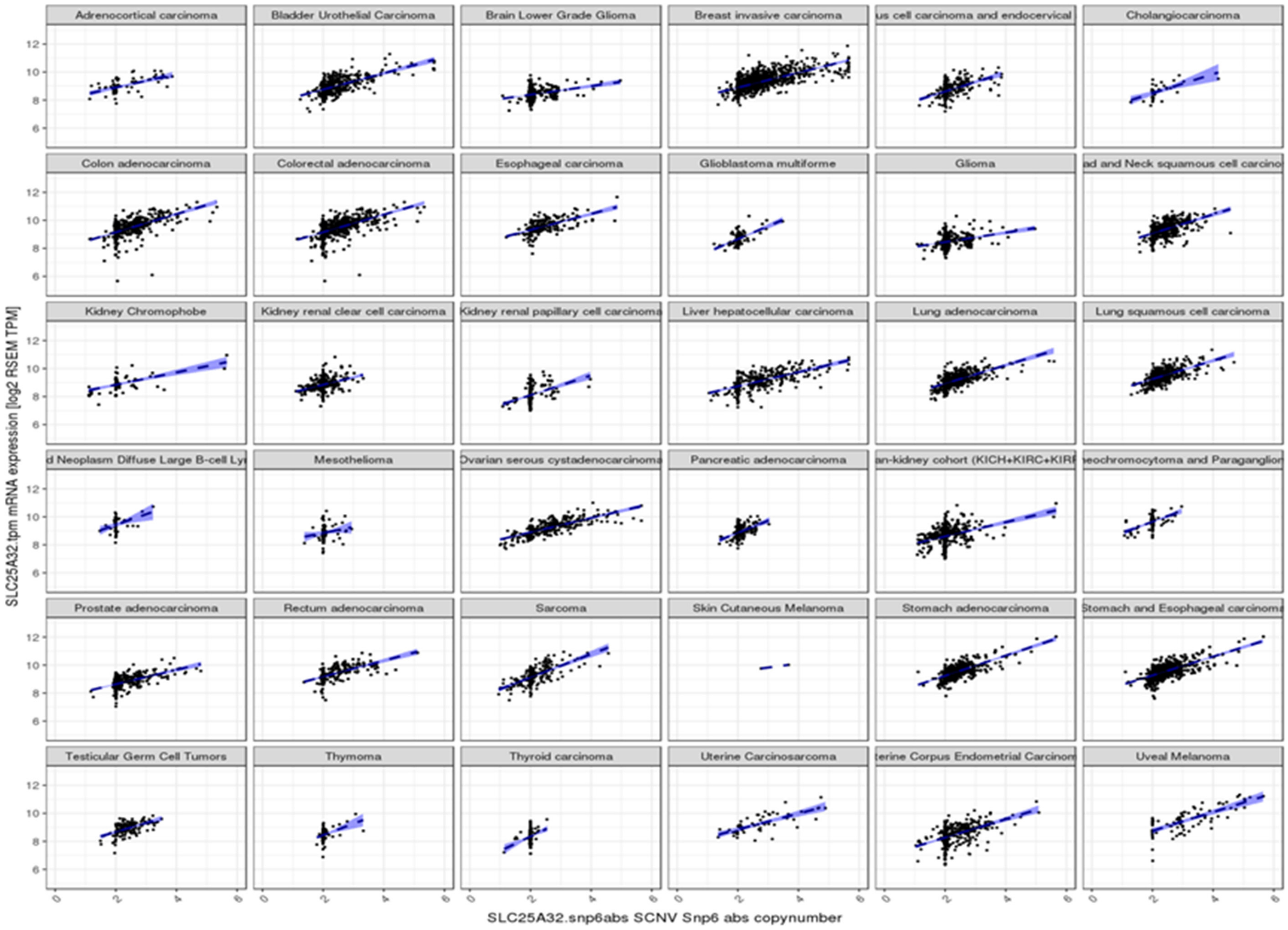

Supplementary Figure 1: SLC25A32 amplification correlates with increased mRNA expression in different tumors. Correlation between SLC25A32 mRNA expression (RSEM TPM) and absolute somatic copy number in patient material of TCGA tumor types. Each dot represents a tumor sample of one particular patient. The dotted line represents a linear regression line and the blue area around the fitted line shows the 95% confidence intervals.

A

Cancer cell Lines	Tissue of origin
MiaPaCa-2	Pancreas
PANC-1	Pancreas
TOV-21G	Ovary
OVCAR-8	Ovary
MDA-MB453	Breast
HCC1143	Breast
AGS	Stomach
DU145	Prostate

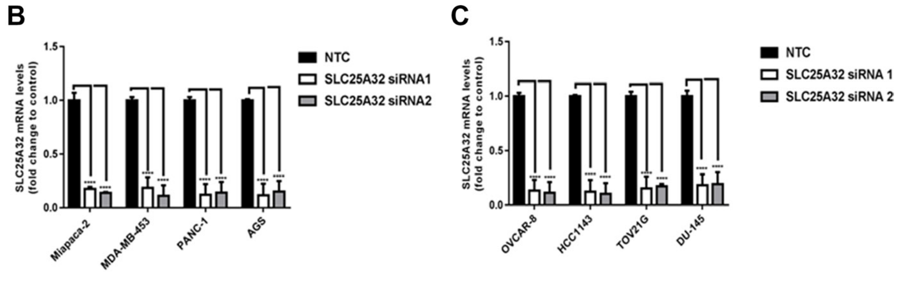

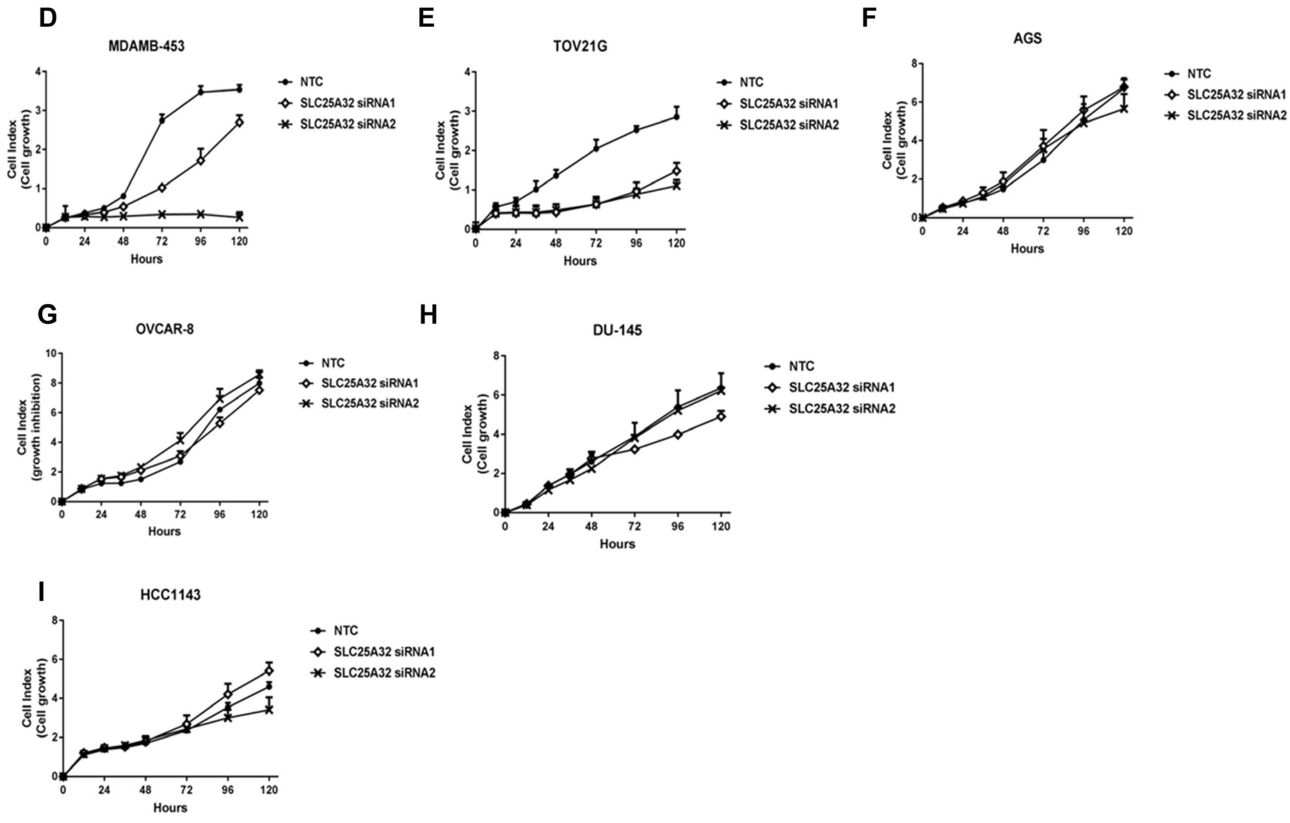

Supplementary Figure 2: Effect of SLC25A32 knock-down on the proliferation of different cancer cell lines. (**A**) Table listing cancer cell lines used in this study and tissue of origin. (**B–C**) Cell lines listed in A were transfected with SLC25A32 siRNA1 and siRNA2 or non-targeting control oligo (NTC) for 72 h. mRNA levels were detected via qRT-PCR and fold change was normalized to NTC sample. HPRT1 gene was used as endogenous control. Results are presented as mean ± SD (*n = 3*). Two-way ANOVA and Bonferroni post-test were used for statistical analysis with Graphpad-Prism6. NTC vs. SLC25A32 siRNA1 or siRNA2, $^{****}P < 0.0001$. (**D–I**) Cells were transfected with either control siRNA oligo (NTC) or SLC25A32 siRNAs (siRNA1 and siRNA2) and real-time cell proliferation of MDA-MB453 (D), TOV21G (E), AGS (F), OVCAR-8 (G), DU-145 (H) and HCC1143 (I) cells was measured with the xCELLigence technology over a period of 5 days**.** Results are presented as mean ± SD (*n* = 3).

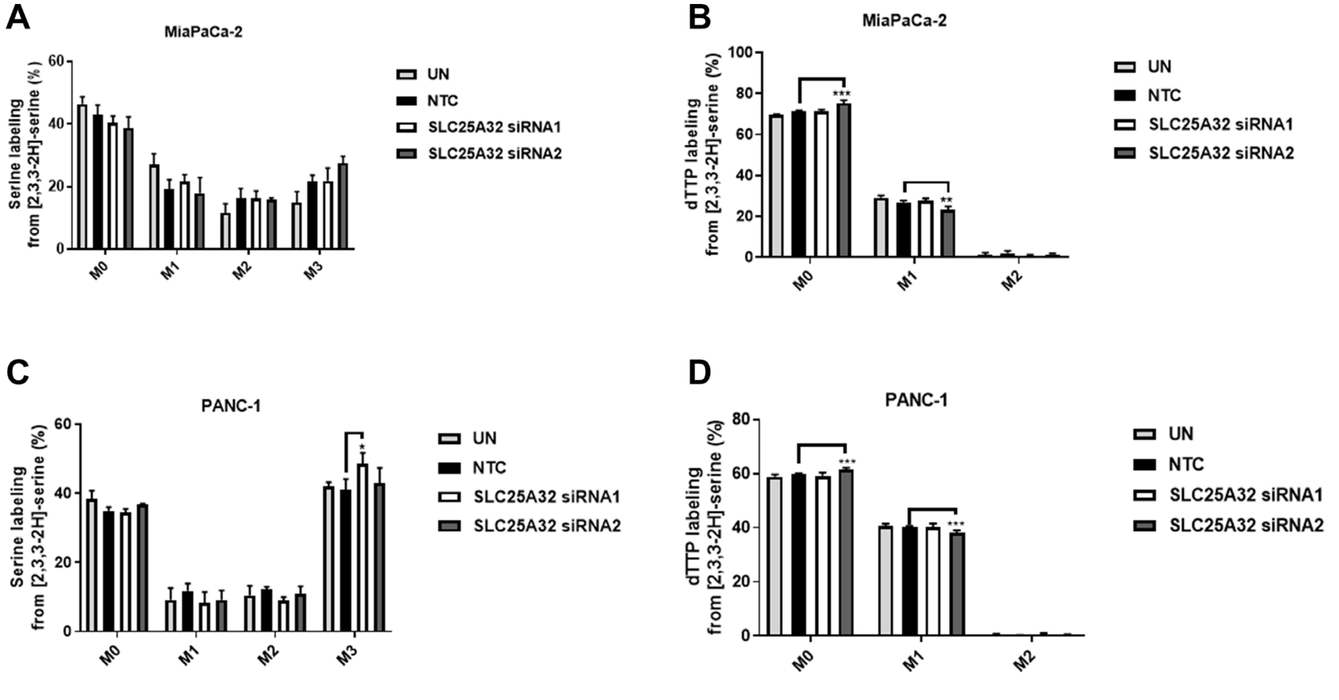

Supplementary Figure 3: SLC25A32 knock-down does not alter the folate cycle flux in MiaPaCa-2 and PANC-1 cells. (**A**) [2,3,3-^{2}H]-serine isotopomer distribution in MiaPaCa-2 cells transfected with either control siRNA oligo (NTC) or SLC25A32 siRNAs (siRNA1 and siRNA2) for 72 h. UN: untreated cells. Results are presented as mean ± SD (*n = 3*). M0, NTC vs. SLC25A32 siRNA2 ($^{***}P = 0.0007$); M1, NTC vs. SLC25A32 siRNA2 ($^{***}P = 0.0033$). (**B**) [2,3,3-^{2}H]-serine labeling into dTTP in MiaPaCa-2 transfected as in A. Results are presented as mean ± SD (*n = 3*). (**C**) [2,3,3-^{2}H]-serine isotopomer distribution in PANC-1 cells transfected as in A. Results are presented as mean ± SD (*n = 3*). M3, NTC vs. SLC25A32 siRNA1 ($^{*}P = 0.0108$). (**D**) [2,3,3-^{2}H]-serine labeling into dTTP in PANC-1 cells transfected with either control siRNA oligo (NTC) or SLC25A32 siRNAs (siRNA1 and siRNA2) for 72 h. Results are presented as mean ± SD (*n = 3*). M0, NTC vs. SLC25A32 siRNA2 ($^{***}P = 0.0010$); M1, NTC vs. SLC25A32 siRNA2 ($^{***}P = 0.0009$) (A–D): Two-way ANOVA and Bonferroni post-test were used for statistical analysis with Graphpad-Prism6.

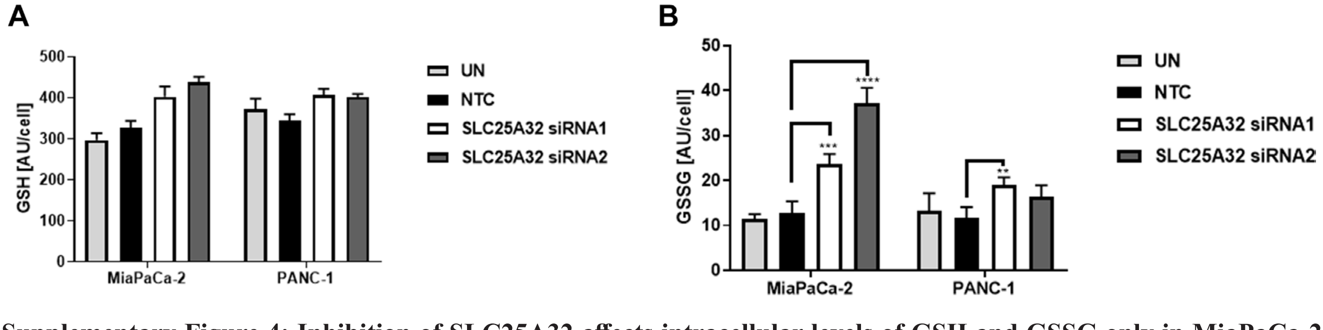

Supplementary Figure 4: Inhibition of SLC25A32 affects intracellular levels of GSH and GSSG only in MiaPaCa-2 cells. (**A**, **B**) MiaPaCa-2 and PANC-1 cells were transfected with either control siRNA oligo (NTC) or SLC25A32 siRNAs (siRNA1 and siRNA2) for 72 h**.** Reduced glutathione (GSH) (A) and oxidized glutathione (GSSG) (B) cellular concentrations were measured by LC-MS. Results were normalized to cell number and presented as mean ± SD (*n = 3*). Two-way ANOVA and Bonferroni post-test were used for statistical analysis with Graphpad-Prism6. MiaPaCa-2, NTC vs. SLC25A32 siRNA1 ($^{***}P = 0.0003$) and NTC vs. SLC25A32 siRNA2 ($^{***}P = 0.0010$). PANC-1, NTC vs. SLC25A42 siRNA1 ($^{**}P = 0.0092$).
